# Predicting Groundwater Indicator Concentration Based on Long Short-Term Memory Neural Network: A Case Study

**DOI:** 10.3390/ijerph192315612

**Published:** 2022-11-24

**Authors:** Chao Liu, Mingshuang Xu, Yufeng Liu, Xuefei Li, Zonglin Pang, Sheng Miao

**Affiliations:** 1 School of Environmental and Municipal Engineering, Qingdao University of Technology, Qingdao 266033, China; 2 School of Information and Control Engineering, Qingdao University of Technology, Qingdao 266033, China

**Keywords:** groundwater quality, deep learning, predictive modeling, LSTM

## Abstract

Prediction of groundwater quality is an essential step for sustainable utilization of water resources. Most of the related research in the study area focuses on water distribution and rational utilization of resources but lacks results on groundwater quality prediction. Therefore, this paper introduces a prediction model of groundwater quality based on a long short-term memory (LSTM) neural network. Based on groundwater monitoring data from October 2000 to October 2014, five indicators were screened as research objects: TDS, fluoride, nitrate, phosphate, and metasilicate. Considering the seasonality of water quality time series data, the LSTM neural network model was used to predict the groundwater index concentrations in the dry and rainy periods. The results suggest the model has high accuracy and can be used to predict groundwater quality. The mean absolute errors (MAEs) of these parameters are, respectively, 0.21, 0.20, 0.17, 0.17, and 0.20. The root mean square errors (RMSEs) are 0.31, 0.29, 0.28, 0.27, and 0.31, respectively. People can be given early warnings and take measures according to the forecast situation. It provides a reference for groundwater management and sustainable utilization in the study area in the future and also provides a new idea for coastal cities with similar hydrogeological conditions.

## 1. Introduction

Urbanization and population growth have increased the consumption of water resources [1]. Water resources are more important for water-scarce areas. With the economic development in recent years, the problems of groundwater pollution and over-exploitation have become more significant. As an important component of water resources, groundwater plays an irreplaceable role in social development, especially in arid and semi-arid regions [2]. The quality of groundwater directly affects the living standard of residents and agricultural development [3]. Many scholars have evaluated the groundwater quality in Nordic and Baltic countries, Korea, and Beijing [4,5,6]. Scholars assessed the quality, groundwater chemical characteristics, and quantity of groundwater by different methods. Affected by geology, pollution indicators, and human factors, the states of groundwater are different. Groundwater resources should be developed and utilized sustainably, avoiding pollution and overuse as much as possible [7].

The long short-term memory (LSTM) neural network is a recurrent neural network (RNN). LSTM is designed to solve the dependency problem of the general neural network [8]. This neural network is widely used in processing the sequence data, such as monitoring data. The regular prediction method are deterministic model, based on the relationship between influencing factors and groundwater quality [9]. Groundwater quality prediction can provide the basis for environmental protection and sustainable development. Different from the traditional prediction methods, this paper combines LSTM to predict the concentration of groundwater indicators.

This paper, based on rainfall and indicator concentration monitoring data, established the LSTM to predict groundwater quality. By collecting monitoring data and monthly rainfall data from October 2000 to October 2014 of Wendeng, the subdistrict of Weihai, the characteristic of groundwater was analyzed. According to the characteristics and groundwater utilization, total dissolved solids (TDS), fluoride, nitrate, phosphate, and metasilicate were screened as characteristic factors. LSTM is established, and the five indicators’ concentrations can be predicted for the dry and rainy periods. Considering the effect of the five indicators on groundwater quality comprehensively, the coastal groundwater quality and changing trend can be assessed and predicted.

The major contributions of this paper are expressed as follows: (1) According to the characteristics of coastal areas, the LSTM established in this paper is used to predict groundwater quality. The basic data used to establish LSTM are previous long-term monitoring data, which overcomes reliance on real-time monitoring data. (2) Combined with prediction data, the changing trend of quality is realized, and protection measures can be taken in advance. (3) The conclusions of this paper can be applied to coastal cities with similar hydrogeological conditions. It provides a basis for the rational use of groundwater resources and the realization of sustainable development.

## 2. Related Works

In arid areas, rainfall is sparse and evaporation rates are high, so groundwater is an important local source of drinking water, for agricultural, industrial, and domestic purposes [10]. Groundwater has less evapotranspiration and is less sensitive to contamination. The challenges to global groundwater supply over the past decade have been enormous. On the one hand, the quantity of groundwater is decreasing [11]. Population growth, urbanization, and excessive extraction of groundwater lead to the water level declining severely [12]. Irrigation using groundwater also decreases the available quantity [13]. On the other hand, the quality of groundwater is worse and worse. Human activities affect groundwater quality [14]. Due to the imperfect sewage treatment facilities, wastewater would pollute the surrounding surface water systems and soil [15,16]. The discharge of domestic wastewater and the excessive use of chemical fertilizers aggravate the pollution of groundwater.

In order to evaluate the pollution degree of the environment, the concentration of factors that caused pollution must be identified [17,18,19]. The groundwater quality index (GWQI) and irrigation water quality index (IWQI) can evaluate groundwater quality for drinking and irrigation purposes [20]. To consider the geographical elements, seepage of surface water pollutants, and other influencing factors, the geographical information system (GIS)-based analytic hierarchy process (AHP) model is applied to predict groundwater quality [21,22].

With the application of computing techniques, different neural network models were developed to evaluate the quantity and quality of groundwater [23,24]. Artificial intelligence (AI) models take advantage of processing nonlinearity data, such as river water quality [25]. Machine learning (ML) models including quantile regression forest (QRF), random forest (RF), radial support vector machine (SVM), stochastic gradient boosting (GBM), and gradient boosting machines are applied to predict water quality (WQ) [26]. Based on the small-scale catchment of Klang River, the novel $H_{2}O$ deep learning (DL) and RF models prediction of river WQI classification is better [27]. Combined with neural network models, the groundwater quality and changing trend would be predicted in advance [28,29]. In particular, artificial neural network (ANN) models have been applied widely in water quality [30].

In the study area, the residents rely on groundwater for subsistence and agricultural needs [31]. However, due to excessive groundwater extraction and agricultural pollution, groundwater quality is under severe pressure [32]. The state and changing trend of groundwater quality would be revealed in advance so that protection measures will be taken. The ion concentration of groundwater is continuous and stable, and LSTM takes advantage of processing sequence data. The ion concentrations can be predicted based on LSTM, providing reference basics for groundwater management.

## 3. Materials and Methods

### 3.1. Study Area

**Figure 1:**
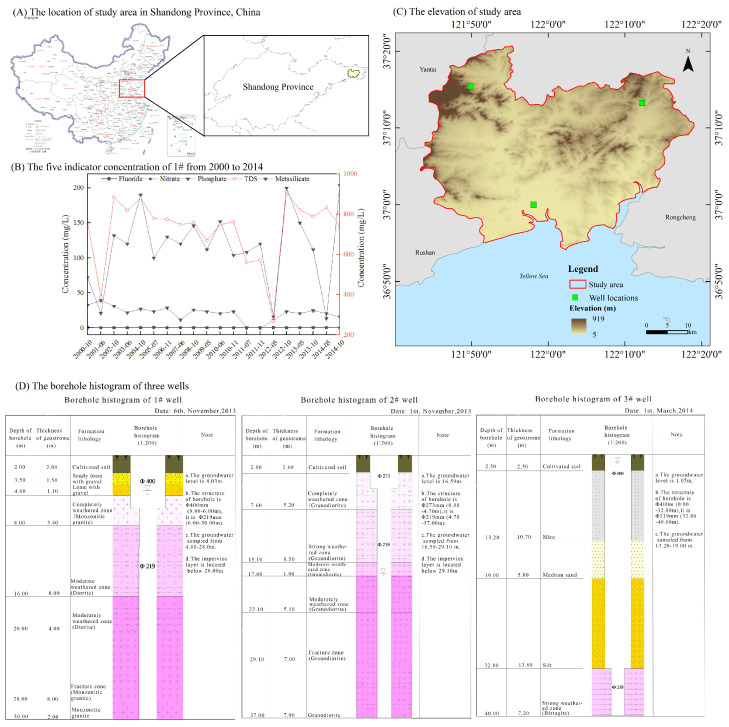
The basic information of study area and three wells.

Wendeng District is bordered by land on the north, west, and east, and by the sea on the south. It is located on the Pacific plate subduction front. The regional tectonic plate is in the SuLu orogenic belt, which is located at the southern end of the Jiaonan-Weihai uplift and Weihai-Rongcheng uplift. From the perspective of stratigraphy, this area belongs to the Ludong stratigraphic division of North China. According to the rainfall and temperature information of the study area, it has a coastline of 156 km with an average annual temperature of 11.5 ଌ and an average annual precipitation of 762.2 mm. The latitude of the study area ranges from 36 degrees to 37 degrees in the northern hemisphere, with a temperate continental monsoon climate. The precipitation is unevenly distributed in the study area, with summer precipitation accounting for about 70% of that annually. The data studied in this paper are based on groundwater monitoring data from three wells. The locations of the study area, the indicator concentrations of well locations, and the borehole histograms are shown in Figure 1.

### 3.2. Index Selection

Due to the shortage of freshwater resources in the study area, the population and industrial and agricultural production are more dependent on groundwater sources. In order to screen indicators on behalf of aspects influencing the quality of groundwater, this paper identifies the beneficial indicators and harmful indicators of water quality.

This paper screened the following indicators to predict and analyze: TDS, fluoride, nitrate, phosphate, and metasilicate. Firstly, dental fluorosis is endemic in around volcanic areas, due to the high fluoride content in daily drinking water. Scholars have analyzed the correlative relationship between dental fluorosis and high fluoride [33]. Secondly, the TDS is screened owing to the agricultural purpose of groundwater in the study area. The study area is coastal, the groundwater depth is shallow, with strong evaporation, TDS will accumulate in the topsoil causing soil salinization [34]. Thirdly, nitrate pollution is caused by the overuse of agricultural nitrogen fertilizers, leaching from municipal landfills, and leakage from industrial wastewater pipelines [35]. According to the International Agency for Cancer Research (IARC), nitrate and nitrite are probable carcinogens [36]. Fourth, according to the news related to the study area, pollution of farmland with superphosphate has occurred. Nitrate and phosphate can assess the impact of urban development and fertilizer use on groundwater quality. Finally, metasilicate is the only positive factor for groundwater quality. According to relevant regulations, when the content of metasilicate is not less than 25 mg/L, it is metasilicate-rich mineral water. As a natural nutritional supplement, metasilicate can help soften blood vessels and promote bone development [37].

### 3.3. Data Sources and Monitoring Methods

In order to demonstrate the seasonal distribution pattern of rainfall and the distribution pattern of dry and rainy periods, the four seasons were used as the time dimension to count the proportion of rainfall in each season to the annual rainfall from 2000 to 2014, and then to account for the proportion of rainfall during the dry and rainy periods. The seasonal and annual variation patterns of precipitation directly determine whether an accurate ion concentration prediction model can be built. Precipitation data are obtained from the long-term monitoring well clusters. Groundwater sampling was carried out once a month according to the operation specification in the Technical Specification for Groundwater Environmental Monitoring (HJ/T164-2004). After determining the groundwater level, washing the well, and stabilizing the parameters, a 500 mL polyethylene sampling bottle was cleaned 2–3 times using the collected water samples and sealed. The ion concentration detection method and standard are shown in Table 1.

### 3.4. Data Preparation

The data are normalized to eliminate the effect of dimensionality between different metrics. Normalized data make the prediction model training results converge to the optimal solution more quickly. Kolmogorov–Smirnov (KS test) and Shapiro–Wilk (SW test) tests are performed by SPSS software. The results are shown in Table 2, the data are less than 0.05, indicating that the data are not a normal distribution. A more suitable normalization method is the min-max normalization. Min-max normalization is characterized by compressing the data interval between [0,1] without changing the characteristics of the data distribution. It is applicable to data sets determined by maximum and minimum values. The calculation formula is as follows:

(1) $$x^{\prime }=\frac{x-x_{\min}}{x_{\max}-x_{\min}}$$

where *x* is the data to be normalized, $x^{\prime }$ is the data from normalized, $x_{\min}$ are the minimum value of data, $x_{\max}$ is the maximum value of data.

A total of 501 data sets were used in this study, taking into account that water quality time series data are generally seasonal, non-linear, and fuzzy, the water quality time series data are divided into rainy period and dry period data. During May to October of each year is the rainy period, and During November to April of the following year is the dry period.

Considering the impact of water quality by this low value, the prediction of the future dry 6 months of the indicator concentration should be based on the past 12 months of monitoring data. In addition, the impact of water quality by rainfall should also be based on the same month of the previous year or years of water quality monitoring data for prediction, so this selection is for the same month for the data of the past two years. Therefore, the input layer of the model is selected for the 12 months before the prediction month and the same months of the previous two years of water quality monitoring data, the output is the water quality data for a month in the dry or rainy season. The monthly data on water quality include five dimensions: TDS, fluoride, nitrate, phosphate, and metasilicate. Therefore, a single sample in the data set is composed of 5-dimensional water quality data for the corresponding 14 months, labeled as the 5-dimensional water quality data of the predicted month.

According to the monitoring data, the three wells are close and the changing trend of indicator concentrations is similar. Therefore, the training set and the test set are established based on the three wells’ monitoring data. After preprocessing the rainfall and monitoring data, 80% of the monitoring data set is randomly screened as the training set and the other 20% as the test set.

### 3.5. Long Short-Term Memory Neural Network

**Figure 2:**
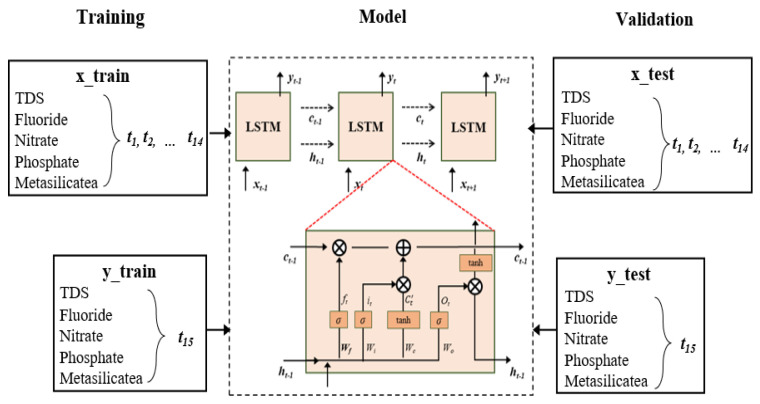
LSTM model structure.

The LSTM model is a gated neural network, and the existence of a “gate” can realize selective memory of information. It is composed of a Sigmoid neural network layer and dot multiplication operation. When the output is 0, no information is passed at this time. If the output is 1, all the information can pass. LSTM model is shown in Figure 2.

(2) $$f_{t}=\sigma \left(W_{f}\left[h_{t-1},x_{t}\right]+b_{f}\right)$$

(3) $$i_{t}=\sigma \left(W_{i}\left[h_{t-1},x_{t}\right]+b_{i}\right)$$

(4) $$C_{t}^{\prime }=\tanh\left(W_{c}\left[h_{t-1},x_{t}\right]+b_{c}\right)$$

(5) $$C_{t}=f_{t}*C_{t-1}+i_{t}*C_{t}^{\prime }$$

(6) $$O_{t}=\sigma \left(W_{o}\left[h_{t-1},x_{t}\right]+b_{o}\right)$$

(7) $$h_{t}=O_{t}*\tanh\left(C_{t}\right)$$

The operating mechanism of LSTM is that with the continuous replacement of data, the model remembers the effective information, forgets the invalid information, and constantly updates the weight status. Each hidden layer of the LSTM model contains three gates (forgetting gate, input gate, and output gate) and a corresponding cell state (C). Firstly, forgetting information is realized by the forgetting gate (F). When the new data $x_{t}$ and the output data $h_{t-1}$ of the last moment enter the model, the forgetting gate processes the data with the help of the Sigmoid function controls the degree of data forgetting and then updates the weight of forgetting gate. The next step is to reach the input gate (I), as shown in Equation (3), the information is processed again with the aim of control that can be stored in the cell state. The degree to which the current calculated state is updated to the cell state can be seen. The cell state at the last moment is multiplied by $f_{t}$ to represent the part to be forgotten, and the new candidate value is represented by $i_{t}*C_{t}{}^{\prime }$, $C_{t}^{\prime }$ to create a new vector of alternative values for a tanh shaped network layer, as in Equation (4). The updated cell state $C_{t}$ is shown in Equation (5). The output gate (O) is then reached, which is used to determine the output content, as shown in Equation (6). The Sigmoid function determines the cell state information to be output, and then the tanh function is used to specify the value between −1 and 1. The tanh adjusted value is then multiplied by the output value as in Equation (7) so that the output value is determined by the model. $W_{f}$, $W_{i}$, $W_{c}$, and $W_{o}$, represent the corresponding weights, *b* represents the corresponding offset term, σ represents the Sigmoid function, and Tanh represents the hyperbolic tangent activation function.

The LSTM model needs to go through two stages before it is put into use: the training stage and the testing stage. The core goal of the training phase is to find the optimal weight. Assuming that there is a certain time sequence data, the data at this time are taken as the target value, the continuous data before this time is used to train the model, and each weight is constantly updated. The training phase ends when the target value is met. In the test phase, the target value is not set, and the error between the output data at the next moment and the real value is checked under the condition of the optimal weight in the current stage. If the error is small, the test phase is over, and the model in this state can be used for this data prediction.

As shown in the training section of Figure 2, the water quality data of TDS, fluoride, nitrate, phosphate, and metasilicate for 14 months are applied in the model used in this paper, and the corresponding month’s (t_15) output water quality data are used to train model. The input of the LSTM model is set to 14 × 5, which corresponds to the five-dimension water quality data received in 14 months. The output is set to 5, which is responsible for the output of the predicted value of five water quality data.

The purpose of LSTM model training is to find a set of optimal model parameters that minimize errors. The water quality prediction model in this paper should be evaluated from the overall performance, hoping that the overall forecast data will be more accurate. Therefore, mean square error (MSE) is selected as the loss function of training. MSE reflects the degree of prediction error through the average sum of the squares of the difference between the predicted data $y^{\prime }$ and the real measured data *y*. Compared with the first-order error loss function MAE, the result is closer to the real situation, which is more sensitive to abnormal outliers and has higher requirements for the overall performance of the model. Finally, the test set is applied to the trained model to get the prediction result of the final model.

(8) $$MSE=\frac{1}{n}{\sum_{t=1}^{n}\left(y-y^{\prime }\right)}^{2}$$

where *y* is the measured value, $y^{\prime }$ are output values.

Taking into account the difference in groundwater quality between the rainy and dry periods, groundwater quality in the rainy and dry periods was predicted with the help of long short-term memory networks based on the time series of water sample data in the study area.

Applying the model to groundwater concentration indicators in the study area requires determining the step size, the number of implied layers, and the number of data dimensions. The concentration of groundwater indicators in the study area is influenced by the amount of rainfall. The annual variation trend is stable. In addition, the variation trends are similar in the dry and rainy periods. Combined with the monitoring data in the study area, the step size of the prediction model is 14.

The prediction accuracy of the model is low, when the number of hidden layers is too small, while the number of hidden layers is too large, it will overfit. After repeated debugging, the final number of hidden layers is one. Combined with the groundwater state of the study area, this paper screened five indicators for prediction, from the perspective of health (TDS and fluoride), agricultural pollution (nitrate and phosphate), and positive index (metasilicate). The number of dimensions of the prediction model is five.

## 4. Results and Discussion

### 4.1. Rainfall Data

The climate type of the study area is a temperate continental monsoon climate. The southeast monsoon blowing from the tropical ocean brings abundant rainfall, there is more rainfall in summer and autumn, while there is less rainfall in winter and spring. The proportion of dry and rainy periods to the total annual rainfall is about 20% and 80%, respectively. The rainfall data are presented in Table 3. Precipitation alternates significantly between rainy and dry periods, with stable water distribution and similar annual distribution. From 2000 to 2014, the proportion of rainfall in rainy and dry periods is essentially constant every year. The stability of seasonal and annual variation is the basis for the concentration prediction model.

### 4.2. Monitoring Results of Groundwater

Affected by surface water infiltrating, the concentrations of groundwater indicators are changing during both rainy and dry periods. The statistics of groundwater indicator concentrations and the longitude and latitude of wells in rainy period are shown in Table 4 and those in dry period are shown in Table 5.

Due to the three wells being close, the changing trends of the indicators are similar. According to Table 4 and Table 5, the average indicator concentration in the dry period is higher than that in the rainy period. Standard deviation and coefficient of variation would measure the statistical dispersion of data, the data in the dry period are higher than that in the rainy period, indicating that the indicator concentrations are more similar in the rainy period.

### 4.3. Modeling Result

**Figure 3:**
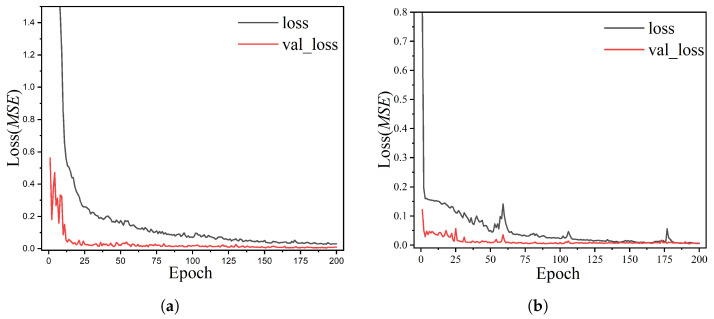
LSTM model training results: (**a**) rainy period; (**b**) dry period.

In order to train and validate the proposed LSTM model, this paper applied TensorFlow 2.6 and Python 3.8 to implement the experiments, Tensorflow is one of the most popular machine learning frameworks available today, it can flexibly create complex topological networks and execute the environment for debugging. The training epoch was set to 50 to achieve a convergent model. Considering that large training epochs can cause undesirable overfitting, this paper employed a model with 200 epochs, as shown in Figure 3. The historical graph of the loss function shows that in the rainy and dry periods, the training set rapidly declines and then slowly converges, while the validation set rapidly declines and then remains stable, and the model converges without overfitting. The models were trained on the two-core Intel(R) Xeon(R) Silver 4210R CPU and NVIDIA GeForce RTX3090 GPU server.

**Figure 4:**
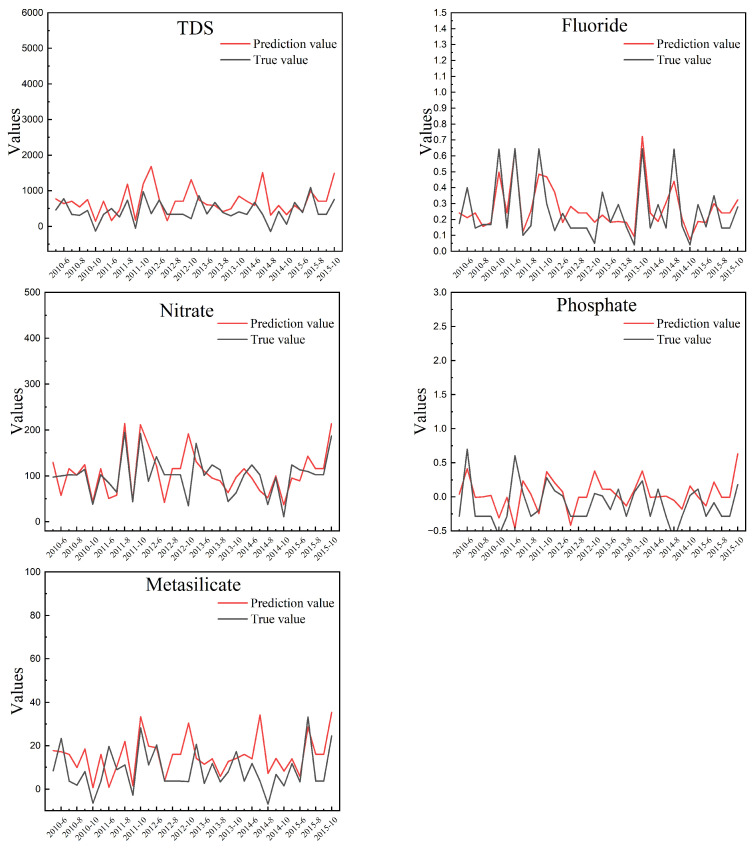
Prediction values and true values for the five indicator concentrations in the rainy period.

**Figure 5:**
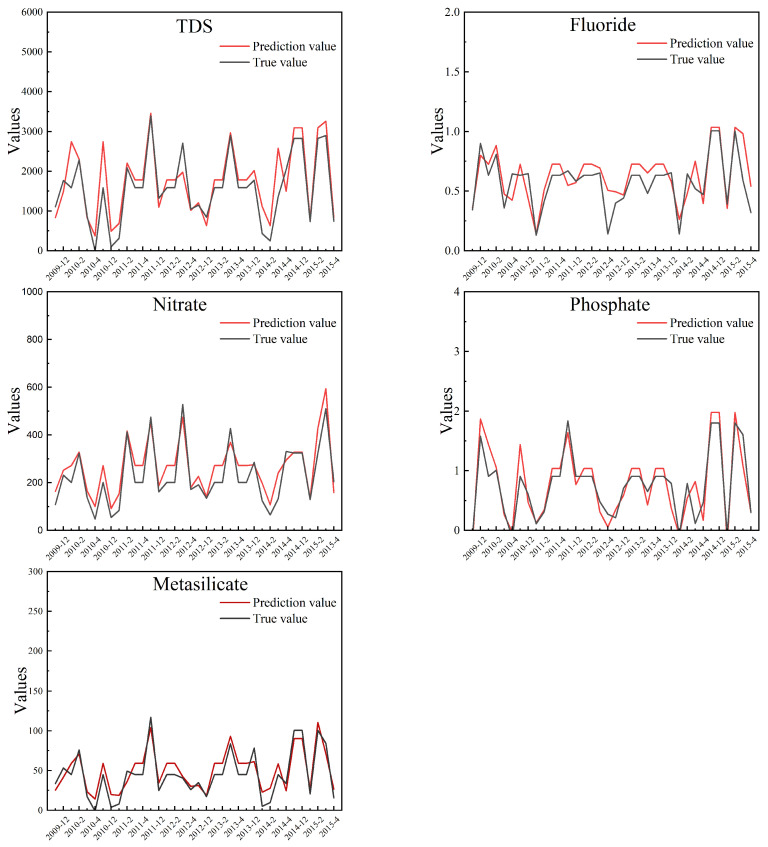
Prediction values and true values for the five indicator concentrations in the dry period.

The predicted values of TDS, fluoride, nitrate, phosphate, and metasilicate in the rainy season are shown in Figure 4. The predicted values of TDS, fluoride, nitrate, phosphate, and metasilicate in the dry period are shown in Figure 5. In the experiment of this paper, MAE is used to measure the error between the real value and the predicted value of the two models. MAE reflects the degree of prediction deviation through the absolute value of the difference between the predicted data and the measured data. This result can reflect the real deviation of five water quality metrics and has realistic evaluation significance. The errors are shown in Table 6.

(9) $$MAE=\frac{1}{n}\sum_{t=1}^{n}\left|y-y^{\prime }\right|$$

According to Table 5, the fitting degree of the five indicators is good, the training of the model for the rainy and dry period has been completed, and the output value of the model can predict the actual concentration. As shown in Figure 4 and Figure 5, the predicted values have similar trends to the true values. In the study area, the true values of the five indicator concentrations changed stably, the concentration in the dry period is higher than that in the wet season. The prediction can reflect the law of change. If the prediction values of TDS are high, it is necessary to pay attention to whether there is a demand for farmland irrigation in the future. Corresponding measures should be taken in time to avoid soil caking caused by slightly saline or saline water, which affects crop yields. If nitrate and phosphate concentrations are predicted to be high, an early warning should be made to note whether the local farming season is approaching and proper planning should be made to avoid excessive use of agricultural fertilizer. When the concentration of metasilicate increases at a certain stable water source point, as a reference, the water source point can be considered as the drinking water source for optimal utilization of the water source.

The model established in this paper can predict groundwater indicator concentration. Compared with traditional methods, such as the MODFLOW, this model does not rely on geological data, and it is simple to carry out. When the indicator concentration data for one consecutive month are available, the model can predict the concentration of the next six months using input data, and the model can continuously output data by circulating input data. The protection measures can be taken in advance, and the predicted values are set as a reference for groundwater protection. Therefore, the model can guide practical work effectively and contribute to the predictability of groundwater quality changes.

### 4.4. Application of the Concentration Prediction

**Figure 6:**
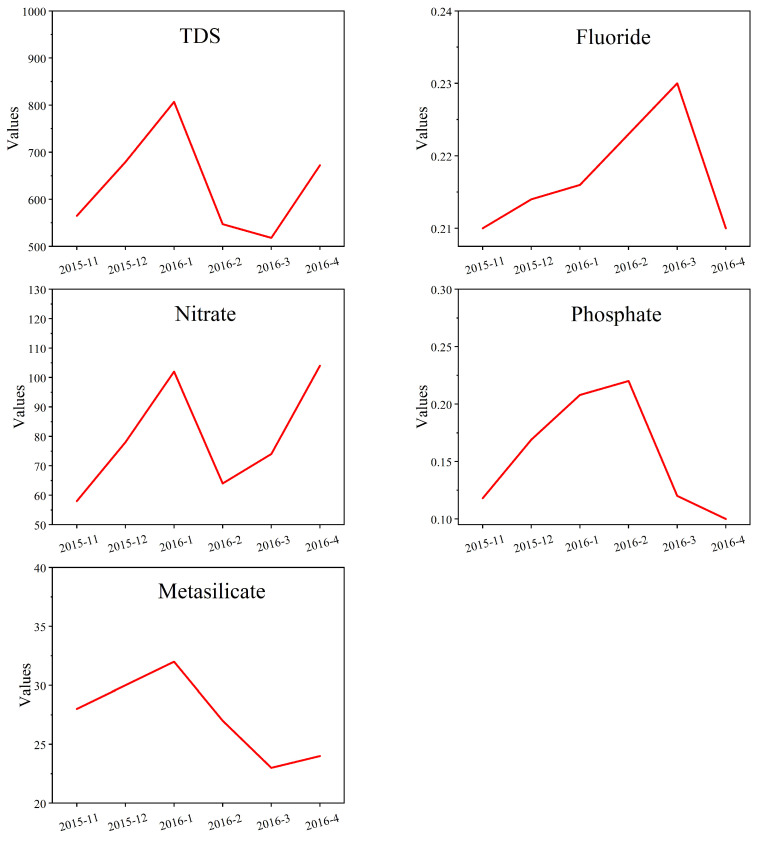
The predicted concentration values of five indicators during the next period.

In this paper, the LSTM neural network model is established and applied to predict the concentrations of five indicators in three specialized observation wells. The prediction values are based on the changing principles of previous indicators’ concentrations, indicating that the model could predict the indicators’ concentrations for the next period. The predicted changing trends of five indicators are shown in Figure 6. From November to February, the concentrations of TDS, nitrate, and metasilicate would fluctuate, and the maximum occurred in February. Due to the quantity of groundwater decreasing, there is less rainwater supply, leading to the concentration increase. The concentration would increase by various degrees from March to April. Compared with TDS and nitrate, the concentration of metasilicate is more stable. The concentrations of fluoride and phosphate would increase from November to March, and decrease from March to April.

## 5. Conclusions

Groundwater, as a main water source, is important for residents’ life. The quality of groundwater plays an irreplaceable role in social development. Groundwater sampling is difficult, causing the monitoring data to not to reflect the current state. Quality prediction results could provide a reference to the administrative department. In this paper, Wendeng District was selected as a study area, and the five indicators were screened, including TDS, fluoride, nitrate, phosphate, and metasilicate. This paper proposed a prediction model based on LSTM, combined with the characteristics of the study area. Based on three wells’ monitoring data over fifteen years, groundwater quality could be predicted. Affected by surface water infiltrating, the concentrations of groundwater indicators change during dry and rainy periods.

In this paper, prediction models of dry and rainy periods were established. MAE is used to measure the errors between the true values and the predicted values. According to the results of LSTM, MAE is low, indicating that the accuracy of the prediction model was high. This model could reflect the groundwater quality changes accurately. Applying the dry period prediction model, the concentration of five indicators was predicted. Prewarning system could be established based on prediction data, abnormal changes will be forecasted. Considering the present and prediction stats of groundwater quality comprehensively, protection measures could be taken in advance.

In future works, more indicators could be taken into consideration, in order to assess groundwater quality more comprehensively. Moreover, the indicators’ concentrations are visible by Arcgis.

## Figures and Tables

**Table 1:** Ion concentration detection methods and standards.

No.	Indicators	Standards	Measurement Method
1	TDS	”Standard examination methods for drinking water—Organoleptic and physical parameters”(GB/T5750.4-2006)	Gravimetric method
2	Fluoride	”Water Quality-Determination of Fluoride-Ion Selective Elec-trode Method”(GB7484-87)	Ion selective electrode method
3	Nitrate	”Water quality—Determination of nitrate-nitrogen—Ultraviolet spectrophotometry” (HJ/T346-2007)	Ultraviolet spectro- photometry
4	Phosphate	”Standard examination methods for drinking water—Nonmental parameters” (GB/T 5750.5-2006)	Molybdenum blue spectrophotometric method
5	Metasilicate	”Drinking natural mineral water test method” (GB8538-2016)	Molybdosilicate blue photometry

**Table 2:** KS test and SW test results of *P* values.

Methods	TDS	Fluoride	Nitrate	Phosphate	Metasilicate
Kolmogorov–Sminov (KS test)	0.030	0.000	0.000	0.000	0.013
Shapiro–Wilk (SW test)	0.000	0.000	0.000	0.000	0.039

**Table 3:** The proportion of rainfall in each wet and dry period from 2000 to 2014.

Year	1#		2#		3#	
	Dry Period	Wet Period	Dry Period	Wet Period	Dry Period	Wet Period
2000	0.20	0.80	0.21	0.79	0.20	0.80
2001	0.24	0.96	0.20	0.80	0.24	0.76
2002	0.16	0.84	0.17	0.83	0.17	0.84
2003	0.21	0.79	0.19	0.81	0.22	0.79
2004	0.15	0.84	0.15	0.85	0.15	0.84
2005	0.16	0.84	0.22	0.78	0.16	0.84
2006	0.14	0.86	0.10	0.89	0.13	0.86
2007	0.05	0.95	0.07	0.93	0.05	0.95
2008	0.29	0.72	0.27	0.73	0.28	0.72
2009	0.21	0.79	0.18	0.82	0.21	0.79
2010	0.08	0.92	0.11	0.89	0.07	0.92
2011	0.27	0.73	0.28	0.72	0.27	0.73
2012	0.20	0.80	0.21	0.78	0.20	0.80
2013	0.25	0.75	0.30	0.70	0.25	0.75
2014	0.26	0.74	0.30	0.71	0.26	0.74
Average	0.19	0.82	0.20	0.80	0.19	0.81

**Table 4:** Statistics of groundwater indicators concentration in the rainy period.

Names of Wells	Geographical Location	Statistical Indicators	TDS (mg/L)	Fluoride (mg/L)	Nitrate (mg/L)	Phosphate (mg/L)	Metasilicate (mg/L)
1	121.831 °E /36.985 °N	Avg Std. CV (%)	421.44 81.02 19.22	93.64 51.05 54.52	0.17 0.12 73.40	0.04 0.03 64.75	30.86 5.11 16.55
2	121.882 °E /37.063 °N	Avg Std. CV (%)	918.52 351.12 38.23	139.60 126.21 90.41	0.18 0.11 63.05	0.09 0.08 94.91	23.75 6.83 28.74
3	121.886 °E /37.093 °N	Avg Std. CV (%)	698.09 196.32 28.12	100.42 60.81 60.55	0.25 0.09 34.51	0.30 0.19 62.23	21.53 7.37 34.23

Abbreviations used in this table include: Avg: average; Std. = standard deviation: CV: coefficient of variation (%).

**Table 5:** Statistics of groundwater indicators in the dry period.

Names of Wells	Longitute/ Latitude	Statistical Indicators	TDS (mg/L)	Fluoride (mg/L)	Nitrate (mg/L)	Phosphate (mg/L)	Metasilicate (mg/L)
1	121.831 °E /36.985 °N	Avg Std. CV (%)	516.90 195.35 37.79	132.22 101.35 76.65	0.25 0.11 44.30	0.04 0.01 40.54	34.84 2.41 6.91
2	121.882 °E /37.063 °N	Avg Std. CV (%)	934.70 359.17 38.43	186.93 135.35 72.41	0.14 0.14 100.56	0.18 0.09 47.17	26.68 4.29 16.08
3	121.886 °E /37.093 °N	Avg Std. CV (%)	764.94 81.64 10.67	138.57 38.90 28.07	0.28 0.21 77.26	0.28 0.14 48.46	26.68 4.71 17.67

Abbreviations used in this table include: Avg: average; Std.: standard deviation; CV: coefficient of variation (%).

**Table 6:** MAE of prediction results based on LSTM.

Indicators (mg/L)	MAE (Rainy Period)	MAE (Dry Period)
TDS	89.45	76.35
Fluoride	0.09	0.06
Nitrate	10.32	5.47
Phosphate	0.23	0.17
Metasilicate	4.21	2.78

## Data Availability

The data presented in this study are available on request from the corresponding author.
